# DOT1L promotes expression of CD44 through the Wnt/β-catenin signaling pathway in early gastric carcinoma

**DOI:** 10.7150/jca.90170

**Published:** 2024-02-25

**Authors:** Ping Li, Zhou Zhang, Ping Sun

**Affiliations:** 1 Department of Pathology, Jiangnan University Medical Center, Wuxi, Jiangsu Province 214002, PR China.; 2 Department of Pathology, Wuxi No.2 People's Hospital, Wuxi, Jiangsu Province 214002, PR China.; 3 Wuxi School of Medicine, Jiangnan University, Wuxi, Jiangsu Province 214002, PR China.; 4 Department of Clinical Laboratory, Affiliated Huishan Hospital of Xinglin College, Nantong University, Wuxi Huishan District People's Hospital, Wuxi, Jiangsu Province 214000, PR China.

**Keywords:** DOT1L, CD44, Wnt/β-catenin, early gastric carcinoma

## Abstract

To assess telomere silencing 1-like (DOTIL) gene expression within gastric cancer (GC) tissues as well as its function of promoting cancer stem cell (CSC)-mediated epithelial-mesenchymal switching, tissue samples from 8 patients each in 3 stages (normal, low-grade intraepithelial neoplasia (LGIN), as well as early gastric carcinoma (EGC)) were collected for whole-exome sequencing, which revealed differentially expressed genes (DEGs). The DEGs and their prognostic value were verified through TCGA and GTEx analyses. We also verified the role of DOT1L in EGC development. We collected samples from three patients each with LGIN and EGC for single-cell sequencing. We conducted single-cell transcriptomic analysis, DEG analysis, cell‒cell interaction analysis, and pseudotime analysis using R language. Sites and levels of DOT1L, CD44 and DOT1L expression were verified by IF. We found 703 deleterious mutation sites in the LGIN group and 389 deleterious mutation sites in the EGC group. The LGIN as well as EGC categories exhibited increased levels of DOT1L expression compared to the standard category (P<0.05) in TCGA and GTEx. DOT1L also correlated significantly with TMB (P=8.45E-06), MSI (P=0.001), and tumor proliferation index (P=7.17E-09) in the TCGA and GTEx datasets. In single cells, we found that DOT1L promotes CD44 expression via the Wnt/β-catenin signaling pathway and the development for stemness properties within GC. In addition, we found that DOT1L, CD44 and CTNNB1 colocalize and correlate positively. In conclusion, one important CSC regulator in GC, DOT1L may be crucial in coordinating the expression of genes specific to a certain lineage during MSC development.

## Introduction

For gastric precancerous lesions, the designations LGIN/dysplasia (LGIN) and high-grade intraepithelial neoplasia/dysplasia (HGIN) are advised, in accordance with the WHO definition of tumours of the digestive system 1. The identification rate of asymptomatic GC or precancerous lesions with early management is increased by improved endoscopy. Japan and South Korea profit through a population-based screening programme since they have lower mortality-to-incidence ratios (0.43/0.35) despite having an elevated incidence 2. Consequently, better outcomes for patients are linked to early detection as well as a proper diagnosis of EGC. A cancer stem cell (CSC) is a subset of cells that still has the ability to differentiate and self-renew 3. Even though CSCs make up a very small percentage for cancer cells, they are known for the capacity to cause tumours, their susceptibility to chemotherapy, and their high propensity for metastasis. These characteristics have been linked to cancer metastasis, recurrence, as well as therapy failure 4, 5. New data has established the presence of CSCs within GC, where they were identified by the production of surface markers characteristic to CSCs 6, 7. Nevertheless, the underlying regulatory mechanism of CSCs in GC remains to be elucidated.

DOT1L represents a highly conserved protein. Studies have revealed that it has a close relationship with gene expression regulation. Based on attribute analysis, it can be seen that it is a methyltransferase (1) (H3K79mex, x = 1, 2, or 3). According to related research, the DOT1L gene can reverse the methylation of histone H3 at position 79; this modification can significantly regulate transcription- and translation-related processes and affect embryonic development. Experimental results show that in ovarian cancer, DOT1L regulates the transcription level of the SPARC gene by methylating the 79th lysine of histone H3 and that the SPARC protein promotes the adhesion of ovarian cancer cells 8; ovarian cancer cell invasiveness and vascular endothelial cell permeability increase when DOT1L expression is elevated. When the expression degree for DOT1L is too high in breast cancer, the transcriptional activity of BCAT is significantly enhanced, which also affects the growth performance for breast tumor cells along with enhances their invasiveness. CSCs aggregate to form microspheres 9, 10, and the DOT1L gene can combine with the c-Myc gene; in this process, mRNA expression of EMT genes is increased, which controls how invasively breast cancer cells can spread. It is unclear how DOT1L contributes to the survival of tumor-initiating or solid stem cell-like cells in tumors.

Numerous biological processes, such as the regulation of the cell cycle, inflammation, cancer, as well as the growth of embryos, depend on the WNT signaling cascade 11. One frequent signaling pathway that is intimately linked to a variety of malignancies is the Wnt signaling system 12-14. The main participant in the Wnt signaling pathway is CTNNB1. Normal circumstances prevent it from building up in the nucleus, which could be crucial in stimulating the TCF/LEF family of transcription factors, which in turn activates genes that respond to Wnt.

Our investigation focused on DOT1L's role in GC cells. The findings provided fresh perspectives into the therapy of GC by demonstrating that DOT1L stimulates the progression of GC via CTNNB1 through upregulating CD44 and activating the Wnt signaling pathway.

## Materials and Methods

### Dataset access and analysis

The Cancer Genome Atlas (TCGA) and Genotype-Tissue Expression (GTEx) were searched **(Table 1)**. The Perl (strawberry-perl-5.32.1.1-64 bit) command was used to convert the genetic probe IDs in the matrix documents to the platform's genetic symbols to acquire a matrix document encompassing formal symbols (https://strawberryperl.com/releases.html strawberryperl.com project). All datasets were normalized using the limma R package (R for Windows 4.1.0). Setup and https://www.rstudio.com). The entire genetic expression dataset underwent log2 transformation. We performed gene differential analysis (|LogFC| > 1, adjusted P value (FDR) < 0.05) by comparing tumor tissues with adjacent healthy controls using the limma R package. Whole-exome sequencing data were analyzed using the Maftools package in R language, and single-cell sequencing data were analyzed using magrittr, dplyr, GSVA, GSEABase, limma, SingleR, and celldex in R language.

### Patient information

The present study was approved by the Ethics Committee of Jiangnan University Medical Center (Wuxi, China; approval no. 20180706). Patients were recruited between January 2020 and December 2022 **(Table 2)**. The inclusion criteria for LGIN patients were as follows: (1) endoscopic mucosal dissection was performed in our hospital, and the postoperative pathology was LGIN; (2) the patient's clinical data were complete, with normal bone marrow function and normal liver and kidney function; (3) the patient had no history of gastrointestinal surgery treatment; and (4) the expected survival period was greater than 6 months. The exclusion criteria for LGIN patients were as follows: (1) the patient had a benign submucosal tumor; (2) the patient had other serious diseases or malignant tumors; (3) the patient had mental illness and weak autonomous expression ability; and (4) the patient was pregnant or lactating. The inclusion criteria for EGC patients were as follows: (1) the patient underwent endoscopic mucosal dissection in our hospital, and the postoperative pathology was EGC; (2) the patient's clinical data were complete, with normal bone marrow function and normal liver and kidney function; (3) the patient had no history of gastrointestinal surgery treatment; and (4) the expected survival period was greater than 6 months. The exclusion criteria for EGC patients were as follows: (1) the patient had other serious diseases or malignant tumors; (2) the patient had advanced gastric cancer or lymph node and organ metastasis; (3) the patient had mental illness and weak autonomous expression ability; and (4) the patient was pregnant or lactating. Written informed consent was obtained for the use of all patient-derived tissues. A total of 8 patients were included. Stomach cancer tissue was collected to assess tumor morphology as part of routine surgery.

### STRING database analysis

Interactions were visualized by Cytoscape version 12.0 (https://cn.string-db.org/), and a PPI network was formed, which was defined as the objective network. Cytoscape is a free software package for visualizing, modeling and analyzing the integration of bimolecular interaction networks with high-throughput expression data and other molecular states 15.

### Immunofluorescence (IF)

Prior to being embedded in paraffin, human tissues have been preserved in 10% neutral-buffered formalin. Utilising Tris/EDTA antigen retrieval buffer, sections had been exposed to antigen retrieval for 20 minutes during 95°C. For IF, sections have been subjected to primary antibodies against CD44 (Abcam, mouse# ab254530; 1:200 dilution), DOT1L (Abcam, rabbit# ab72454; 1:100 dilution) and CTNNB1 (Abcam, mouse#ab22656; 1:100 dilution) during 4°C overnight, after which the goat anti-mouse IgG HL (Alexa Fluor 488 1:100 dilution) as well as goat anti-IgG HL (Alexa Fluor 594 1:100 dilution) were incubated with fluorophore-conjugated secondary antibodies. For thirty minutes, DAPI (Beyotime, P0131) was utilised during room temperature. The sections have been examined using an Olympus fluorescence microscope.

### Whole-exome sequencing

Through informatics examination of high-throughput sequencing download files, genetic information about the human whole-genome sequence can be retrieved. Quality assurance, initial processing of off-board data, as well as statistical sequencing information output all had been incorporated within the bioinformatics investigation; BWA 16 along with additional applications were used to compare the information to the reference genome. GATK 17 software was used for variant detection. SNPs and InDels were annotated using ANNOVAR 18 software.

Single-nucleotide variants (SNVs) are the most common type of human variation, which refers to the variation of a single base in the genome.

Significantly changed genes were found using the MutSigCV algorithm, version 1.41 (significance set at Q < 0.05). The Control-FREEC method, version 11.1, was utilised to identify somatic copy number alterations (SCNAs). The HDGC cohort's frequently amplified or deletion genomic areas were found using the GISTIC2.0 algorithm 19. In accordance with the frequency and magnitude of amplification or deletions within each gene, G scores have been determined across sequencing regions. A heterozygous germline mutation that turned homozygous was referred to as a double-hit event. This could increase the effect of germline variations and possibly be linked to carcinogenesis. More information on the methodologies section includes information on target capture sequencing, double-hit event evaluation, drug target examination, and the discovery of somatic single-nucleotide variants (SSNVs), indels, along with SCNAs.

### Single‐cell RNA‐seq using 10x Genomics Chromium

Single-cell RNA and TCR sequencing: We collected six gastric tissue biopsy specimens. PBMCs that had just been extracted were used to create single-cell libraries utilising 10X Genomics DNA V3 Reagent Kits. Gel beads bearing barcoded oligonucleotides (UMIs) as well as oligo dTs have been combined with the cells with kit reagents to create reaction vesicles known as gel bead-in-emulsions (GEMs). GEM-RT was supplemented with primers against common 5' along with 3' ends to amplify completely barcoded cDNA using PCR. Enough material has been generated by this amplifying reaction to create several libraries—including T-cell-enriched and 5' gene expression libraries—from the same sample. Illumina-ready sequencing libraries were generated using the Single Cell 3' Protocol. Illumina-ready sequencing libraries that are V(D)J-enriched and 5' gene expression have been created using the Single Cell V(D)J Reagent Kit methodology. Gene Denovo Biotechnology Co. conducted libraries' sequencing on the Illumina HiSeq 4000 platform with a successful concentration (Guangzhou, China).

### Cell-cell interaction prediction in single-cell transcriptomics data

Using the CellPhoneDB programme (version 2.0.0), cell-cell interactions between all kinds of cells have been estimated using single-cell RNA sequencing information 20. Merely ligand-receptor relationships with a P value <0.05 have been utilized to forecast cell-cell relationships in the cell types, and the mean of every one of the partner average expression values within the associated interaction pairs among various kinds of cells has been contrasted.

### Statistical analysis

Investigation of single-cell information: Using the human reference version GRCh38 as a guide, Cell Ranger Pipeline (https://github.com/10XGenomics/cellranger) produced raw gene expression matrices for every sample. R software was used to analyze the output filtered gene expression matrices using the Seurat tool 21. Filtering cells based on gene expression, unique UMI, as well as proportions of mitochondrial genes served as the initial step in the quality control process. The R software was used to convert and normalize the information for each sample in order to eliminate ambient contamination. Moreover, doublets within every sample were eliminated using DoubletFinder. The samples had a batch impact, as demonstrated by PCA. Accurate integration of single-cell information from many technological systems as well as batches is possible with the Harmony algorithm 22. As a result, information from several samples evaluated by the Harmony algorithm was combined. Then, grouped cells were visualized in two dimensions using the PCA, tSNE, as well as UMAP clustering methods. The top 5 genes were displayed within a heatmap utilising the Do-Heatmap function, and DEGs have been identified utilising the FindAllMarkers function. The R package clusterProfiler has been employed to carry out the gene enrichment analysis 23. SCENIC (single-cell regulatory network inference and clustering) has been employed to conduct the TF study.

Using the TCGA database (https://portal.gdc.cancer.gov/), RNA-sequencing expression (level 3) identities, genetic mutations, along with associated clinical information have been obtained for DOT1L. The R software's maftools package was used to obtain and visualize the mutation information. The histogram displays genes whose mutational frequency was significantly greater within stomach cancer individuals compared with healthy controls. *P < 0.05 has been recognized as a statistically significant value.

## Results

### Somatic alteration landscape of stomach tissue in 8 patients

**Figure 1:**
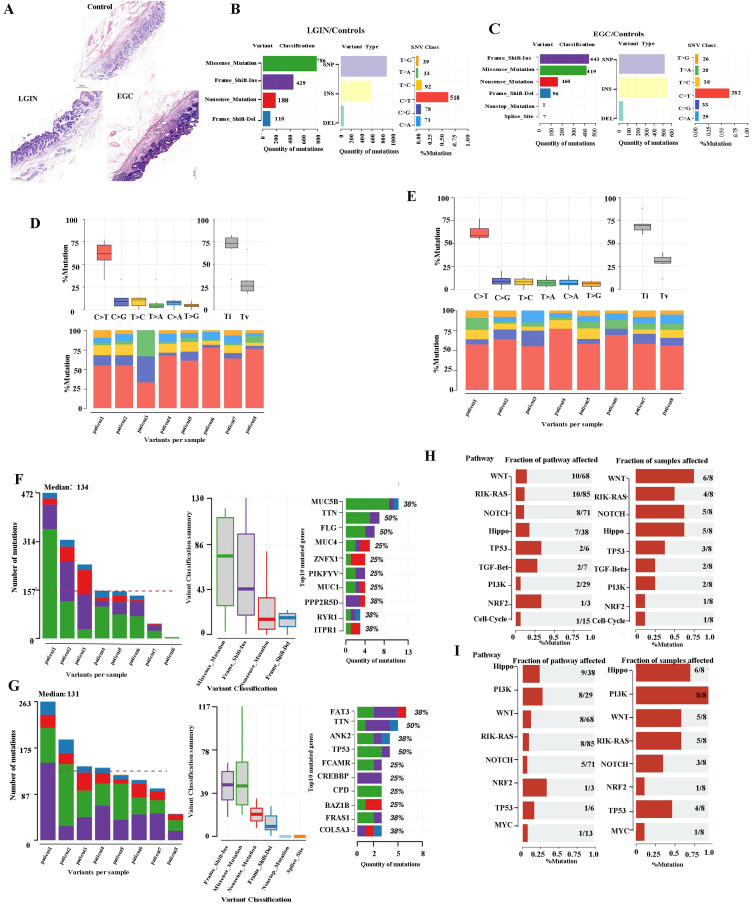
** Somatic alteration landscape of stomach tissue in 8 patients. A.** Eight specimens were matched and detected by WES at 3 stages (normal, LGIN, EGC). **B.** Variant classification in the LGIN group. The X-axis and Y-axis represent the number of variants and variant classification, respectively. variant type in the LGIN group. The X-axis and Y-axis represent the number of variants and variant type, respectively. SNV class in the LGIN group. The X-axis and Y-axis represent the ratio and SNV class, respectively. The number on the right represents the number of SNV classes. **C.** Variant classification in the EGC group. The X-axis and Y-axis represent the number of variants and variant classification, respectively. variant type in the EGC group. The X-axis and Y-axis represent the number of variants and variant type, respectively. SNV class in the EGC group. The X-axis and Y-axis represent the ratio and SNV class, respectively. The number on the right represents the number of SNV classes. **D.** Analysis of the difference in SNV class in the LGIN group. **E.** Analysis of the difference in SNV class in the EGC group. **F.** Variants per sample in the LGIN group. The X-axis and Y-axis represent the sample and number of variants, respectively. Top 10 mutated genes in the LGIN group. The X-axis and Y-axis represent the number of variants and mutated genes, respectively. The number on the right represents the percentage of mutated genes. **G.** Variant classification summary in the EGC group. The X-axis and Y-axis represent the variant classification and number of variant classifications, respectively. Top 10 mutated genes in the EGC group. The X-axis and Y-axis represent the number of variants and mutated genes, respectively. The number on the right represents the percentage of mutated genes. **H.** Fraction of pathways affected and fraction of samples affected in the LGIN group. **I** Fraction of pathways affected and fraction of samples affected in the EGC group.

We used whole-exome sequencing for 24 endoscopic submucosal dissection (ESD) tissue specimens from 8 patients (Fig. 1A). The top 10 mutant genes are displayed in the stacked bar graph. We assessed mutations in the LGIN group that were not present in the normal group. Dominant mutations were missense mutations (786). Single-nucleotide polymorphisms (SNPs), specifically C>T or T>C mutation, were the dominant common mutation subtype (Fig. 1B). Frame-shift insertion (443) was the greatest prevalent variation type in the EGC group. The particularly prevalent SNP genotype was the C>T or T>C mutation (Fig. 1C). C>T was the main type of base change in both the LGIN and EGC groups (Fig. 1D, E).

The number of changed bases within each sample seemed considerably greater within the LGIN category compared with the EGC category, but no changes in categorization were seen among the two categories (Fig. 1D, E). We analyzed each sample to determine tumor heterogeneity (Fig. 1F, G). The LGIN and EGC groups showed the top 10 mutant genes. There was a substantial difference within the mutation rates of the top 10 driver genes among the LGIN as well as EGC categories. The top 10 driver genes for the LGIN group (MUC5B, TTN, FLG, MUC4, ZNFX1, PIKFYVE, MUC16, PPP2R5D, RYR1, and ITPR1) are shown in Fig. 1F. The top 10 driver genes for the EGC group (FAT3, TTN, ANK2, TP53, FCAMR, CREBBP, CPD, BAZ1B, FRAS1, COL5A3) are shown in Fig. 1G. According to the fraction of pathways affected and samples affected, the WNT, RIK-RAS, NOTCH, Hippo, TP53, TGF-Beta, PI3K, NRF2, and Cell Cycle pathways were affected in the LGIN group (Fig. 1H) and the Hippo, PI3K, WNT, RTK-RAS, NOTCH, NRF2, TP53, and MYC pathways in the EGC group (Fig. 1I). Genes were mainly enriched in the Hippo, RTK-RAS, Wnt, NRF2, NOTCH, and PI3K signaling pathways in LGIN or EGC. The Wnt signaling pathway was differentially enriched in 6/8 samples in the LGIN group and 5/8 samples in the EGC group.

### TCGA database validation of gastric cancer mutation maps

**Figure 2:**
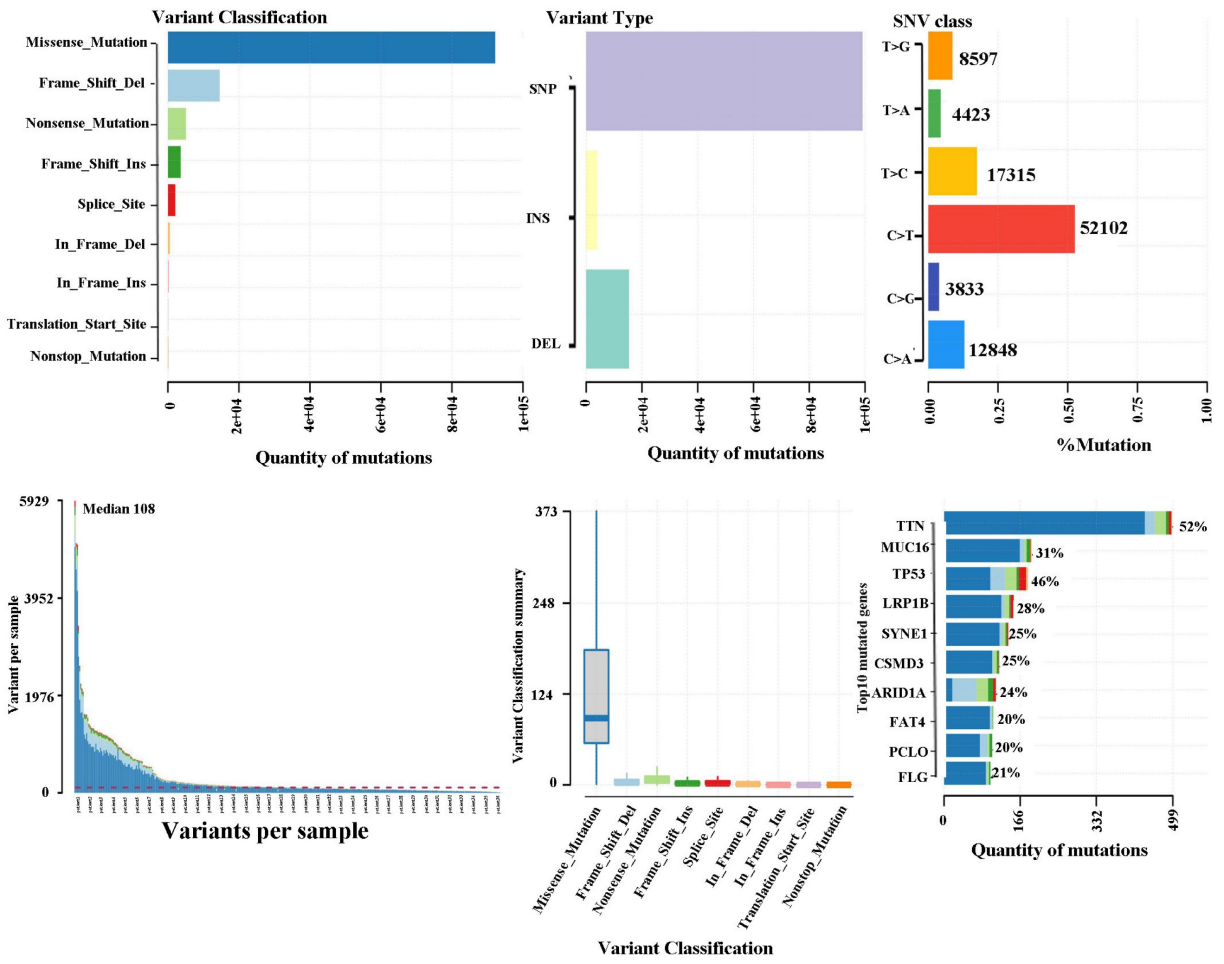
** TCGA database validation of gastric cancer mutation maps.** Oncoplot shows the somatic landscape of the stomach tumor cohort in the TCGA database.

The somatic landscape for the cohort with stomach tumours is displayed on Oncoplot. The distribution of variations by variation categorization, kind, as well as SNV category can be seen within the cohort summary graphic. The mutation load of each sample (variant categorization type) is shown within the bottom section, which reads through left to right. The top 10 mutant genes are displayed in a stacked bar graph (Fig. 2). According to the TCGA database, missense mutations constituted the most prevalent variation for stomach cancer. SNPs represented the most common mutation category when looking at mutation types. The most prevalent SNP type has been the C>T transition. There were 108 variants in the median. The top 10 mutated genes (TTN, MUC16, TP53, LRP1B, SYNE1, CSMD3, ARID1A, FAT4, PCLO, and FLG) are shown.

### Identification of hub genes in stomach cancer

**Figure 3:**
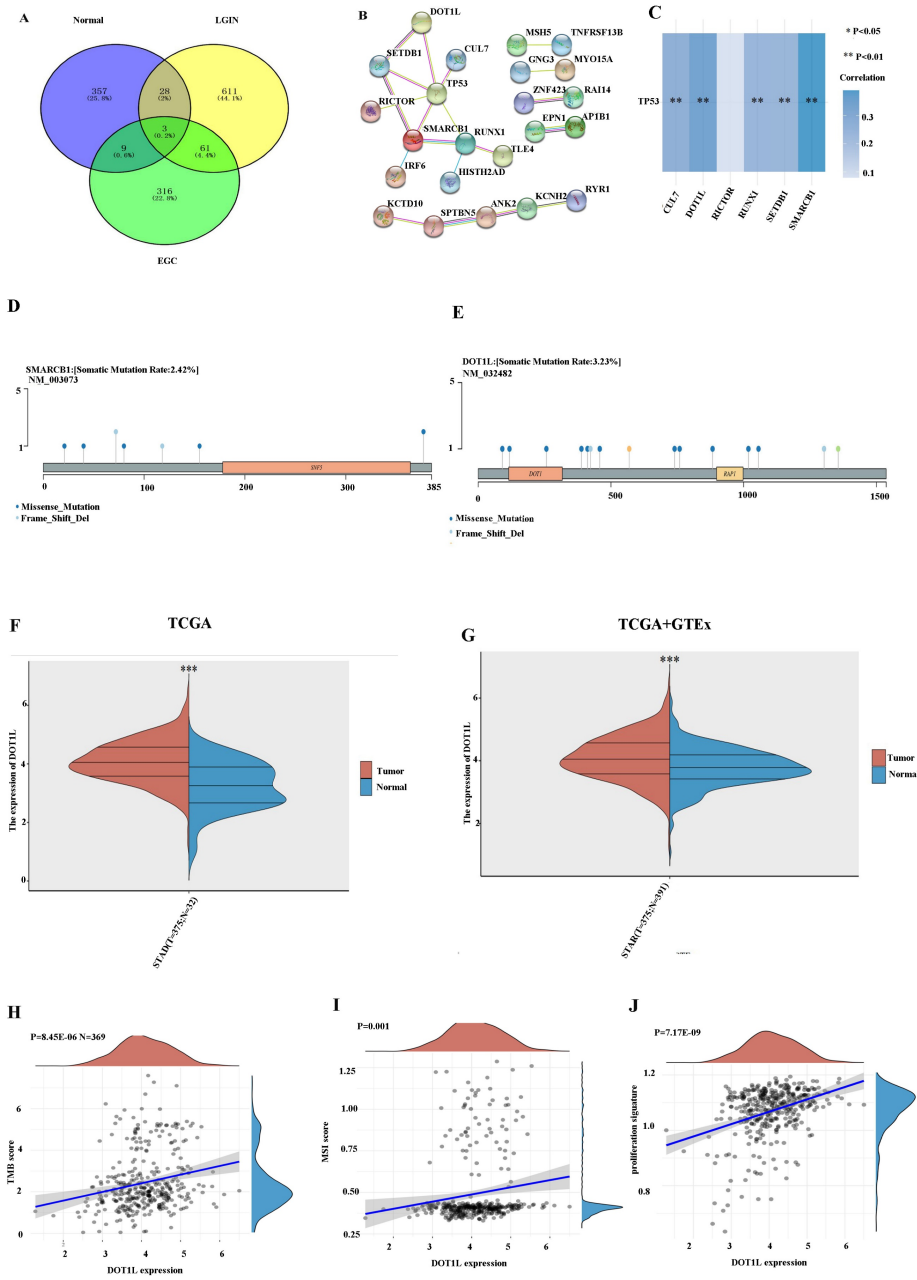
** Selection of differentially expressed genes. A.** A total of 61 (4.4%) genes were chosen in the LGIN and EGC groups but not in the normal group by the Venn program. **B.** STRING was used to construct and visualize the PPI network of DEGs. **C.** The correlation between six genes and TP53. **D.** Lollipop charts show that the somatic mutation rate of the SMARCB1 gene is 2.42%. **E.** Lollipop charts show that the somatic mutation rate of the DOT1L gene is 3.23%. **F.** Expression of DOT1L in 375 tumor patients was significantly higher than that in 32 normal controls from the TCGA database. **G.** Expression of DOT1L in 375 tumor patients was significantly higher than that in 391 normal patients from the TCGA and GTEx databases. **H.** Expression of DOT1L correlated positively with TMB. **I.** Expression of DOT1L correlated positively with MSI. **J.** Expression of DOT1L correlated positively with cell proliferation.

We found that 61 (4.4%) genes were expressed in both the LGIN and EGC groups but not in the normal group (Fig. 3A). The PPI network for DEGs was built along with visualised using these genes (Fig. 3B). By observing the top 10 mutant genes in TCGA data and WES, we found that only TTN and TP53 appeared in these two groups at the same time (Figs. 1, 2). It has been established that TP53, one of the frequently occurring tumour suppressor genes in human tumours, is crucial to the onset and progression of GC 24. First, we chose genes interacting with TP53 using the STRING website; the detailed results are shown in Fig. 3B. Six genes (DOT1L, SMARCB1, RUNX1, CUL7, SETDB1, and RICTOR) closely linked to TP53 were identified. Through TCGA data analysis, among the six gene groups, DOT1L and SMARCB1 had the closest positive correlation with TP53 (Fig. 3C). Lollipop charts for the DOT1L gene mutation; Fig. 3D displays the name of the somatic mutation as well as the somatic mutation rate of 3.23% within the figure caption. Lollipop charts of the mutant SMARCB1 gene; the names for the somatic mutations are displayed within the subheadings, while the figure caption displays the somatic mutation percentage, which is 2.42% (Fig. 3E). The dataset under analysis comprised 32 nearby normal tissues as well as 375 individuals with stomach cancer. The TCGA database showed that the expression for DOT1L within 375 tumour individuals remained substantially greater compared to that within 32 normal controls (Fig. 3F). To avoid the small number of normal TCGA groups affecting the results, we again used the TCGA and GTEx databases for verification. According to the TCGA and GTEx datasets, the expression for DOT1L within 375 tumour individuals were substantially in excess of within 391 healthy individuals (Fig. 3G). We chose the TCGA database to verify the association among DOT1L expression and prognosis along with found that expression for DOT1L correlated positively with TMB (Fig. 3H), MSI (Fig. 3I), and cell proliferation (Fig. 3J). We speculate that expression of DOT1L affects tumor prognosis.

### Workflow for analyzing the stomach atlas and scRNA-seq profiles of stomach tissue

**Figure 4:**
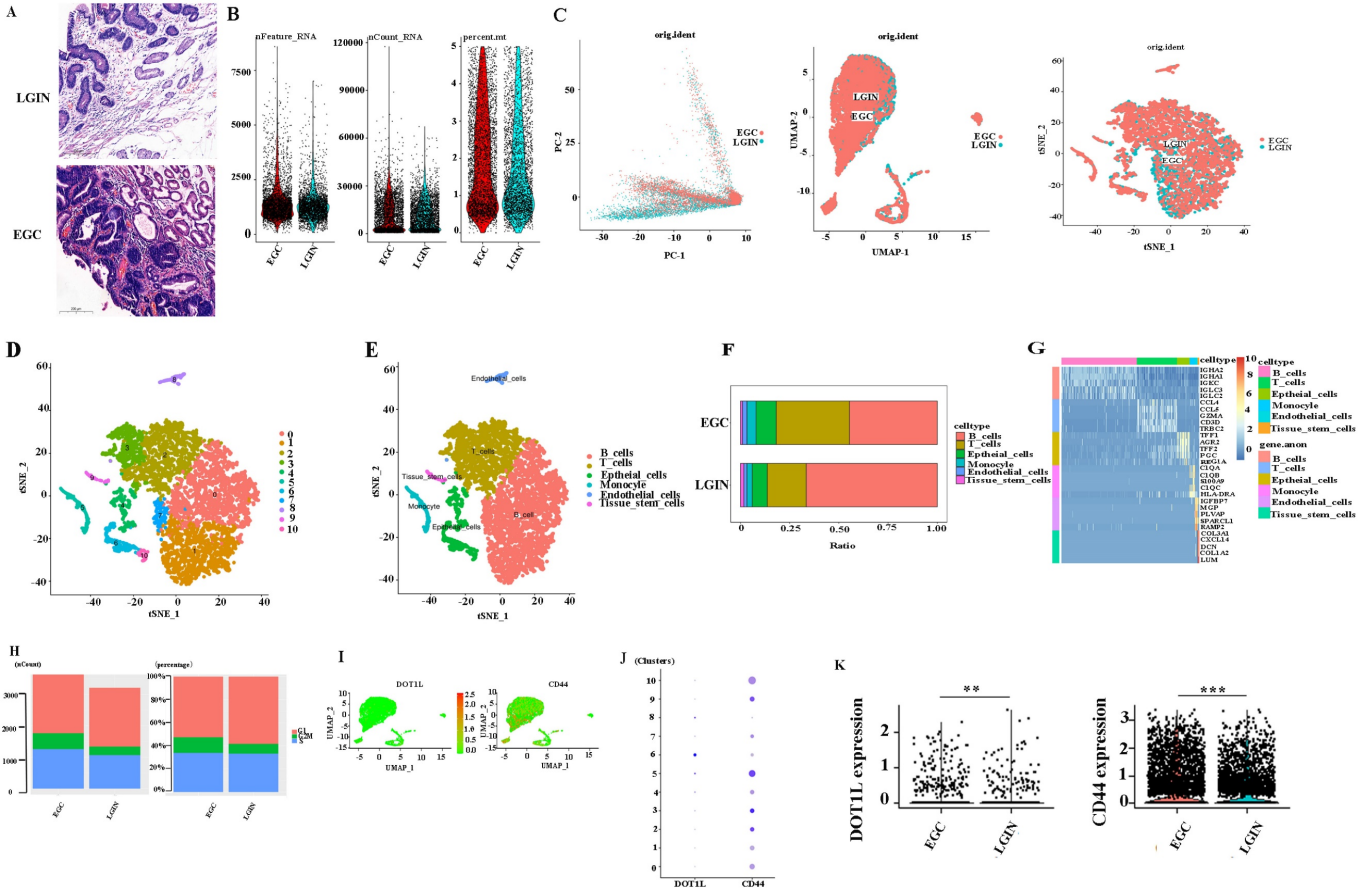
** Single-cell transcriptome profiling of PBMCs in all samples. A.** Project landscape. PBMCs of 3 LGINs and 3 EGCs. Six groups of specimens were matched from 3 patients at two periods (LGIN and EGC). **B.** Quality control of single-cell data by R packages. **C.** Similar PCA, umap, t-SNE plot of different cell types colored according to individual stomach samples (EGC and LGIN). **D.** t-SNE plot of different origins, clusters and cell types in stomach samples. **E, F.** The relative proportion of each cell type in all stomach tissues is shown. **G.** The top 5 DEGs were visualized in a heatmap using the Do-Heatmap function. **H.** We calculated and represented the number and proportion of cell cycle activity (G1, G2/M, S group) in LGIN and EGC. **I, J, K.** We observed expression of DOT1L and CD44 in the LGIN and EGC groups.

To determine the expression and mechanism of action of DOT1L (Fig. 4A), Six different surgical tissue specimens from three individuals were used to build single-cell RNA-seq (scRNA-seq) libraries, which produced expression profiles for 6931 (EGC: 3719, LRIN: 3212) individual cells. The range of nFeature_RNA was 71 to 8588, and the median was 1253. The range of nCount_ RNA was 500 to 117567, and the median was 9427. The range of percent.mt was 0 to 4.996773, and the median was 1.580006 (Fig. 4B). Consequently, information from several samples evaluated by the Harmony algorithm was combined (Fig. 4C). Then, the Seurat package visualised grouped cells in 2D space in LGIN and EGC using the PCA, tSNE, and UMAP grouping methods 25 (Fig. 4D, E). Cell clustering analysis with Seurat 25 using 6931 cells revealed six distinct cell populations, which represented B cells (clusters 0, 1, 7) and T cells (clusters 2, 3), epithelial cells (clusters 4, 6, 10), monocytes (cluster 5), endothelial cells (cluster 8), and tissue stem cells (cluster 9) (Fig. 4F). Consistent with previous findings, B cells (LGIN: 66.6%; EGC: 44.6%) and T cells (LGIN: 19.8%; EGC: 37.2%) constituted a significantly greater fraction of the tumour microenvironment. In contrast, epithelial cells (LGIN: 7.7%; EGC: 10.2%), monocytes (LGIN: 2.6%; EGC: 4.6%), endothelial cells (LGIN: 1.6%; EGC: 2.3%), and tissue stem cells (LGIN: 1.7%; EGC: 1.0%) accounted for a smaller proportion, probably a reflection of the change towards immune cells that invade tumours (Fig. 4G). The top 5 genes have been shown as a heatmap utilising the Do-Heatmap operation, while DEGs were obtained utilising the FindAllMarkers service (Fig. 4H). Compared with the LGIN group, there were more G2/M stages and relatively few G1 stages in the EGC group. The aforementioned findings further supported the validity of the tissue selection by showing that the EGC group had stronger cell division. We first analyzed the expression and distribution of DOT1L and CD44. The proportion of cells expressed is indicated by the dot size, as well as the relative expression level is indicated by the colour intensity (Fig. 4I, J). We then evaluated expression of CD44 and DOT1L in ten clusters. DOT1L was highly expressed within clusters 5, 6, and 8. CD44 has been highly expressed within clusters 2, 3, 5, 9, and 10. These 5 clusters represent epithelial cells. We speculate that DOT1L and CD44 can be coexpressed in epithelial cells (Fig. 4K). We observed expression of DOT1L and CD44 in the LGIN and EGC groups and found that expression of both CD44 (P<0.001) and DOT1L differed in these groups (P<0.001).

### Location and expression of CD44 and DOT1L in stomach tissue

**Figure 5:**
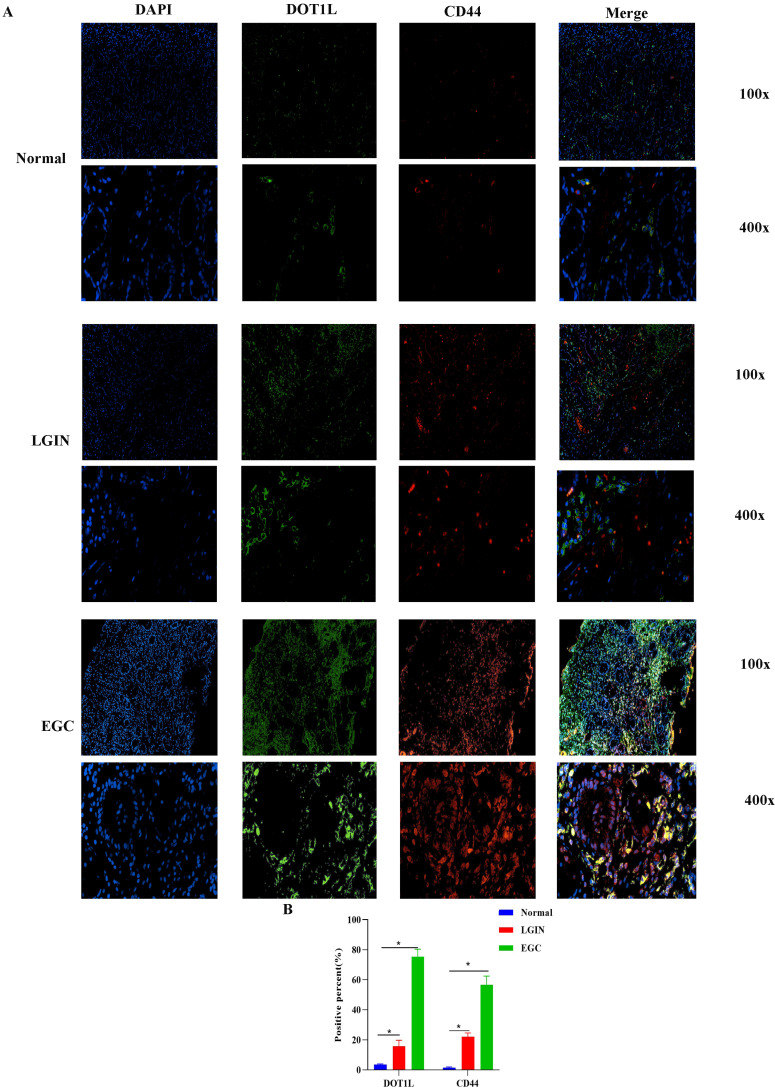
** The location of DOT1L and CD44 expression was determined in stomach tissue. A.** Colocalization and expression of DOT1L and CD44 in stomach tissue by IF. **B.** Quantitative analysis of IF staining.

To determine expression of DOT1L and CD44, we performed IF coexpression (Fig. 5A). DOT1L-positive reactants were in the nucleus of cells. CD44-positive reactants were in the membrane of cells, as shown in Fig. 5. IF analysis of stomach cancer tissue revealed colocalization of CD44 and DOT1L, as shown in Fig. 5A. Expression of DOT1L and CD44 in LGIN and EGC was considerably greater compared to normal controls (P<0.05) (Fig.5B).

### Cell-cell communication network among different cell types in stomach tissues

**Figure 6:**
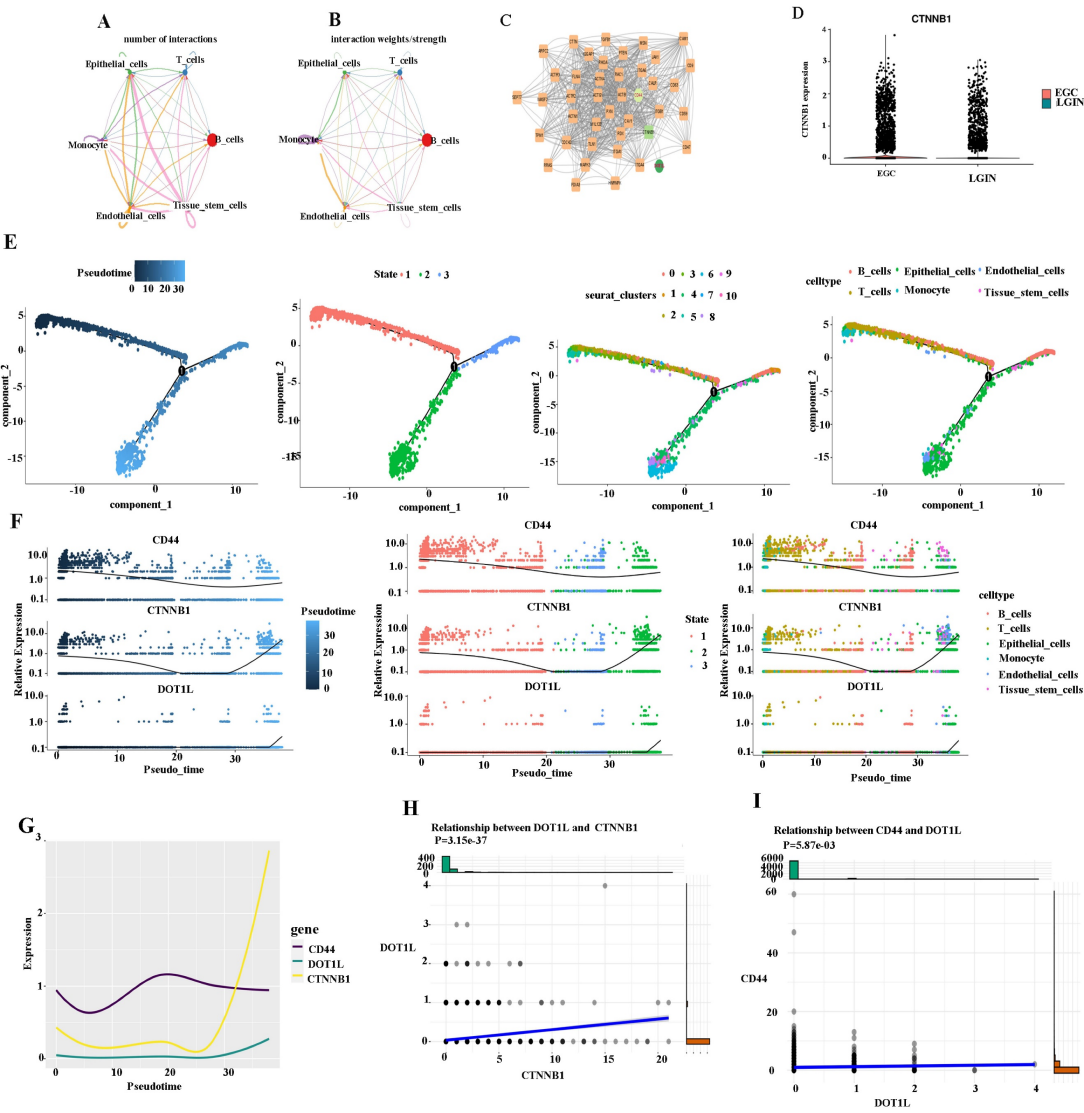
** Cell-cell communication network among different cell types in LGIN and EGC. A, B.** The cell-cell communication network of the number of interactions among different cell types in stomach tissue. The cell-cell communication network of interaction weights/strength among different cell types in stomach tissue. **C.** DEGs compared between different cell types in different groups by the Seurat package. **D.** We observed expression of CTNNB1 in the LGIN and EGC groups. **E.** Pseudotime trajectory analysis was used to analyze the progression of continuous cell states of stomach tissue and revealed ordered cells expressing different levels of marker genes in a trajectory. **F.** We examined changes in genes as a result of cell trajectories. **G.** CD44, CTNNB1 and DOT1L showed the same trend, and both showed an upward trend in epithelial cells. **H.** CTNBB1 correlated positively with DOT1L (P=3.15e-37). **I.** CD44 correlated positively with DOT1L (P=5.87e-3).

We created a cell-cell communication network based on the quantity and strength of connections between various cell types in the primary stomach to ascertain the mechanism of action between CD44 and DOT1L (Fig. 6A). The largest number of connections was observed between epithelial cells and tissue stem cells. However, monocytes were closely linked to tissue stem cells from the aspect of interaction weights/strength (Fig. 6B). DEGs were compared between different cell types in different groups by the Seurat package (Fig. 6C). DEGs were identified using Cytoscape 26. We found that the CD44 gene is closely linked to the DOT1L gene through the CTNNB1 gene. We suspect that DOT1L may affect WNT signaling by influencing expression of CTNNB1 and promote the formation of cancer stem cells by promoting CD44 expression (Fig. 6C). We observed expression of CTNNB1 in the LGIN and EGC groups (Fig. 6D). We present pseudotime trajectory analysis through four modes (pseudotime, state, Seurat_clusters, and cell type). The majority of T, B, and epithelial cells were placed within a major trajectory with two bifurcations according to the pseudotime ordering for cells. Cancer cells as well as tissue have been identified towards the other end of the trajectory, while epithelial cells had been found towards the trajectory's origin, which partially acted as confirmation for the trajectory's construction (Fig. 6E). According to changes in genes as a result of cell trajectories, CD44, CTNNB1 and DOT1L showed the same trend, upward, in epithelial cells (Fig. 6F, G). CTNBB1 correlated positively with DOT1L (P=3.15e-37) (Fig. 6H) and CD44 with DOT1L (P=5.87e-3) (Fig. 6I).

### Cell-cell communication network among different cell types in the LGIN and EGC groups

**Figure 7:**
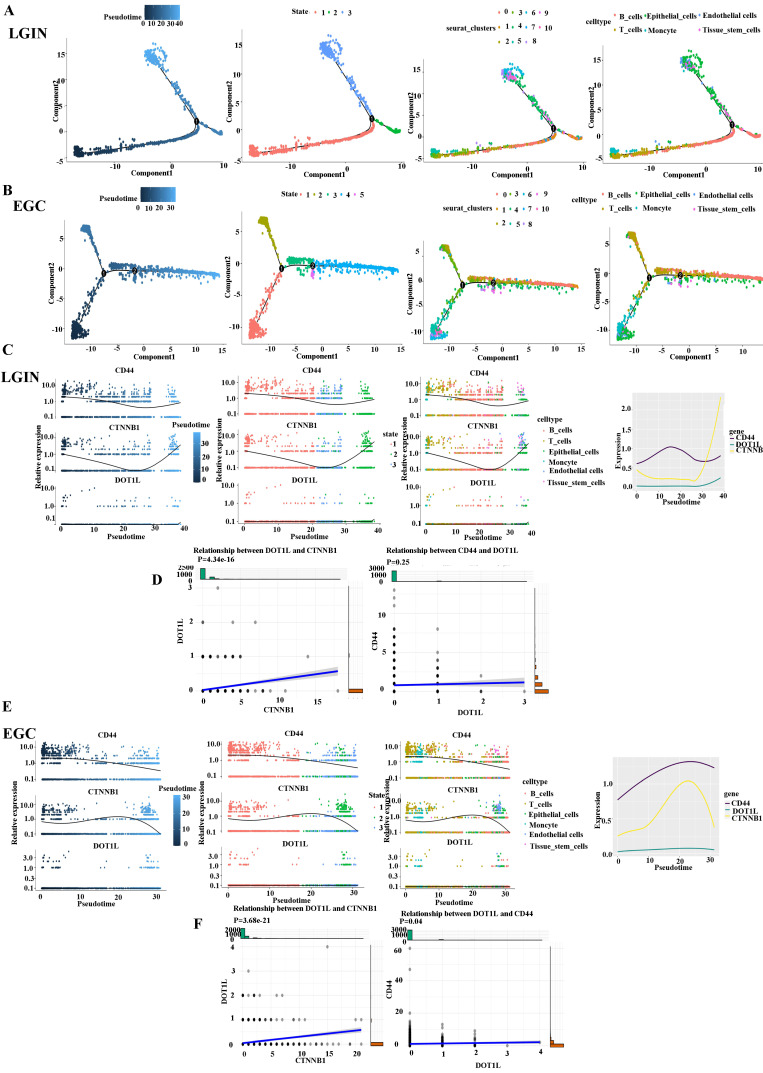
** Cell-cell communication network among different cell types in the LGIN and EGC groups. A.** Pseudotime trajectory evaluation has been utilised to examine the evolution of continuous cell states in stomach tissue in the LGIN group in order to ascertain the trajectory of cell differentiation in the LGIN (3212) and EGC (3719) groups. **B.** The EGC group's stomach tissue's continuous cell state evolution was examined using pseudotime trajectory assessment. **C.** Pseudotime trajectory analysis in the LGIN group demonstrated ordered cells expressing various quantities of marker genes in a trajectory. In the LGIN group, CD44, DOT1L and CTNNB1 showed a trend of first decreasing and then increasing. **D.** In the LGIN group, CTNBB1 was positively correlated with DOT1L. Although CD44 and DOT1L showed the same trend, CD44 and DOT1L did not show a positive correlation (P=0.25). **E.** Pseudotime trajectory research in the EGC group demonstrated ordered cells expressing various quantities of marker genes in a trajectory. In the EGC group, CD44, DOT1L and CTNNB1 showed a trend of first decreasing and then increasing. Compared to the EGC group, CD44 and DOT1L expression was found to be stronger over time in the LGIN group. **F.** In the EGC group, CTNBB1 correlated positively with DOT1L. In the EGC group, CD44 correlated positively with DOT1L.

We divided stomach tissue into two groups, LGIN (3212) and EGC (3719), to determine the trajectory of cell differentiation between the groups (Fig. 7A). We present pseudotime trajectory analysis through four modes (pseudotime, state, Seurat_clusters, and cell type) (Fig. 7A, B). Pseudotime trajectory analysis in the LGIN group demonstrated ordered cells expressing various quantities of marker genes within a trajectory (Fig. 7C). In the LGIN group, CD44, DOT1L and CTNNB1 showed a trend of first decreasing and then increasing. In the LGIN group, CTNBB1 correlated positively with DOT1L (P=4.34e-16). Although CD44 and DOT1L exhibited the same trend, they did not show a positive correlation (P=0.25) (Fig. 7D). Pseudotime trajectory research within the EGC category demonstrated ordered cells expressing various quantities for marker genes within a trajectory (Fig. 7E). In the EGC group, CD44, DOT1L and CTNNB1 showed a decreasing trend. Compared to the EGC group, CD44 and DOT1L expression was found to be stronger over time in the LGIN group. In the EGC group, CTNBB1 correlated positively with DOT1L (P=3.68e-21); in the EGC group, CD44 correlated positively with DOT1L (P=0.04) (Fig. 7F).

### Location and expression of DOT1L and CTNNB1 in stomach tissue

**Figure 8:**
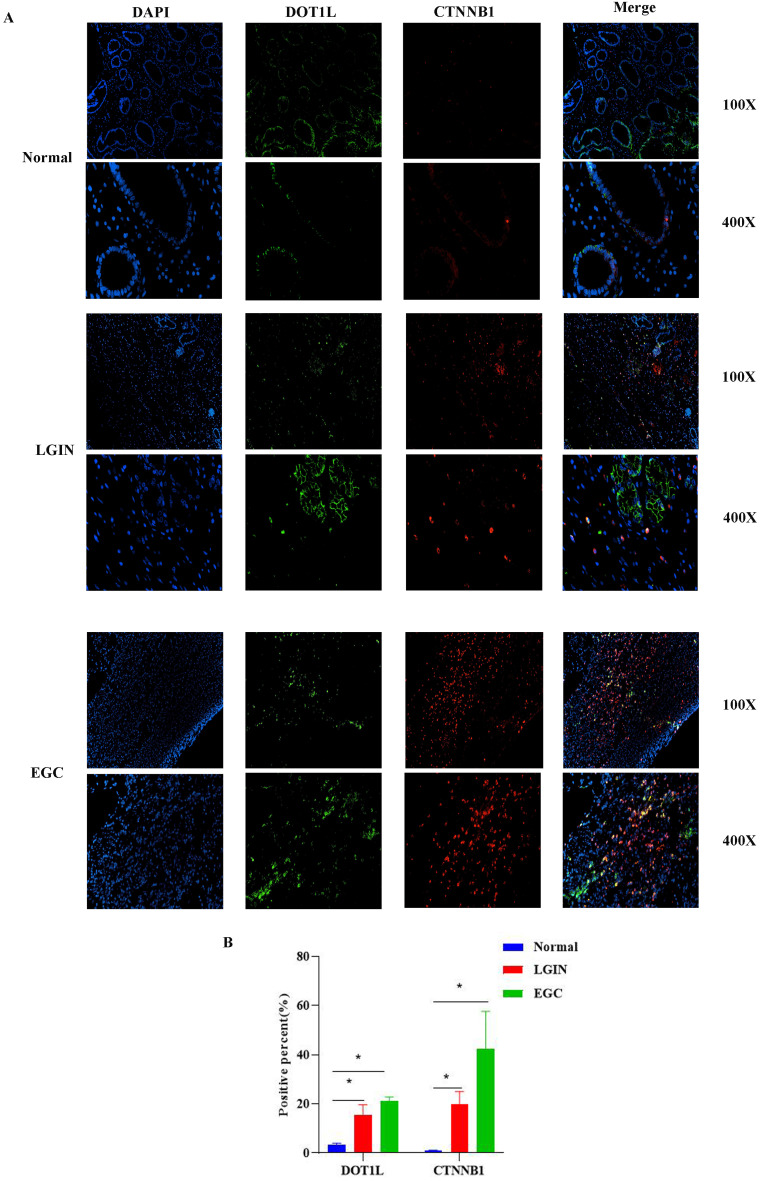
** The location of DOT1L and CTNNB1 expression was determined in stomach tissue. A.** Colocalization and expression of DOT1L and CTNNB1 was determined by IF in normal, LGIN and EGC tissues. **B.** Quantitative analysis of IF staining.

Colocalization of CTNNB1 and DOT1L was used to determine whether DOT1L expression can act on CTNNB1 in stomach tissue (Fig. 8)**.** IF analysis of stomach cancer tissue revealed colocalization of CTNNB1 and DOT1L, as shown in Fig. 8A. CTNNB1-positive reactants were detected in the cytoplasm of cells, as shown in Fig. 8. In comparison to normal controls, LGIN and EGC had substantially greater levels of DOT1L and CTNNB1 expression (P<0.05) (Fig.8B).

## Discussion

GC is a highly prevalent cancer globally, with the second-highest incidence along with death rates among all cancer types. When the age of onset was examined, it was shown that young individual's incidence of GC continues to rise 27. Individuals with GC had "three strong and three poor" traits: high incidence, mortality, and metastatic rates, and poor rates of early detection, radical resection, as well as 5-year survival 28. Ninety percent of EGC sufferers survive five years after undergoing major surgery and chemotherapy 28. Consequently, it is very important to search for biological indicators related to GC and to detect EGC via tumor markers.

With the great improvement of computer data processing capabilities, bioinformatics 29 has made a major breakthrough with the help of big data, with great achievements in research on tumors, especially molecular biology mechanisms. With the advent of precision medicine 30, basic research on the molecular biology of tumor mechanisms is particularly important. Whole-exome sequencing is a crucial genomics research technique and instrument that can be used to selectively sequence the coding portions of the human genome. This allows researchers to identify aberrant genes linked to both common and uncommon disorders. Whole-exome sequencing has a high sensitivity 31. In this study, tissue samples from 8 patients at 3 stages (normal stage, LGIN and EGC) were collected, and whole-exome sequencing was performed on 24 samples. The study's findings indicate that there may be some variations among LGIN as well as EGC categories in comparison to the normal category, but the mutation type and mutation ratio are very similar, which also shows that cancer is an evolutionary process, and each stage has similarities and differences with other stages.

We found 703 deleterious mutation sites in the LGIN group and 389 deleterious mutation sites in the EGC group. We selected DOTIL, which was not expressed in the normal group but was expressed in both the LGIN group and the EGC group, for analysis. High expression of the DOT1L gene can promote tumor progression, and studies on related prostate cancer have also obtained the same results 32, 33. We used both TCGA and GTEx data to verify results. Compared to that in normal tissue, expression of DOTIL was significantly increased in EGC and LGIN tissue.

The advent of high-throughput scRNA-seq research has allowed for the unprecedented delineation of many complex biological reactions. This has been especially helpful for organogenesis studies because scRNA-seq has a strong capacity to dissect cell heterogeneity in complex tissues 34. We investigated the precise mechanism for action of DOT1L within LGIN as well as EGC using scRNA-seq. By analysing scRNA-seq information, a tiny percentage of tissue stem cells was discovered in the LGIN and EGC categories. Because of their capacity for self-renewal as well as multidirectional distinction, CSCs, a relatively tiny subset of cancer cells living within the TME, are thought to play a role in tumour start, heterogeneity, propagation, and therapeutic resistance 35-37.

According to the CSC hypothesis and studies of other solid tumors, CSCs are highly tumorigenic and have multidirectional differentiation ability and chemotherapy/radiotherapy resistance 38. They are self-renewing and can produce many heterogeneous tumor cells, which are closely related to the occurrence, recurrence and metastasis of tumors. Many studies have demonstrated that CD44 serves as a marker for several tumor stem cells, including gastric CSCs 39-42. Expression of CD44 mRNA is significantly higher in gastric CSCs than in a GC cell line 39, 43. CSCs hijack properties unique to normal tissue stem/progenitor cells to enable tumorigenesis and therapy resistance. To target CSCs, we need to better understand the mechanisms that sustain them. Through pseudotime trajectory analysis, we found that stem cells mainly appear at tissue intersections, and stem cells can be used as a major factor to promote the occurrence of EGC.

Oncogenic activation of DOT1L has been implicated in several cancers. In MLL (MLL-rearranged AML), DOT1L represents a therapeutic target 44, 45. Constitutive DOT1L recruitment is caused by MLL1 translocations with several gene partners, activating genes essential to leukemogenesis 46. Prognostic and therapeutic consequences of DOT1L overexpression within ovary 47 as well as malignancies of the colon 48, breast 49, 50 and neuroblastoma 51 are known. CTNNB1 is a kind of mRNA that can be translated into a protein connected with the Wnt signaling pathway 52. Research has demonstrated that slow nonatrophic gastritis, chronic atrophic gastritis, intestinal metaplasia, dysplasia, gastric cancer, and other diseases are accompanied by a progressive rise in positive expression of CD44 53. This implies that CD44 can be utilised as a diagnostic standard for precancerous lesions since it is thought to be an oncogenic gene that contributes significantly to the early degradation of the gastric mucosa. In our study, expression of DOT1L correlated positively with that of CD44 and CTNNB1 according to single-cell sequencing analysis of the EGC group. In the LGIN group, CD44, DOT1L and CTNNB1 showed a trend of first decreasing and then increasing. In the LGIN group, CTNBB1 correlated positively with DOT1L. Although CD44 and DOT1L showed the same trend, a positive correlation was not observed. We hypothesize that the low number of cells may be due to the lack of a positive correlation between DOT1L and CD44 in the LGIN group. We used both IF datasets to verify that CD44, DOT1L and CTNNB1 showed a trend of first decreasing and then increasing. However, due to the difficulty of using endoscopes to obtain human specimens involved in this study, the number of specimens obtained was relatively small. In the future, we will expand the number of specimens in this study to make the results more scientific and universal.

Our hypothesis is that the expression of DOT1L rises as the disease worsens and that it has a positive correlation with the expression of CD44. By encouraging the production of CD44 via the Wnt/β-catenin signaling pathway, DOT1L helps GC develop stem cell-like characteristics. The process for the action of CSCs is intricate, as well as future research will examine the connection among DOT1L as well as the Wnt signaling pathway.

## Figures and Tables

**Table 1:** Clinical information of gastric carcinoma patients from the TCGA and GTEx database

Clinical features	Number (TCGA)	Number (GTEx)
**Gender**		
Female	139	128
Male	236	263
**Age**		
20-39	4	180
39-59	107	52
>60	264	159
**Survival Status**		
Dead	150	
Alive	224	
Unknown	1	
**M**		
M0	337	
M1	24	
Unknown	14	
**N**		
N0	112	
N1	100	
N2	74	
N3	72	
Unknown	17	
**T**		
T1	21	
T2	83	
T3	166	
T4	97	
Unknown	8	
**Stage**		
I	53	
II	112	
III	152	
IV	35	
Unknown	23	
**All**	375	391

**Table 2:** Clinical information of patients from our hospital

Clinical features	Number
**Gender**	
Female	3
Male	5
**Age**	
20-39	1
39-59	3
>60	4
**Helicobacter pylori**	
Positive	4
Negative	4
**Stage**	
I	3
II	3
III	1
IV	1
**All**	8
